# Association Between Venous Thromboembolism Prophylaxis and the Incidence of Thromboembolism Following Orthopedic Procedures: A Cross-Sectional Study

**DOI:** 10.7759/cureus.52652

**Published:** 2024-01-21

**Authors:** Moula Ghulam, Zarbakhta Ashfaq, Saad Ali, Ahad Nawaz, Nouman Anthony, Usman Ghani, Omer Farooq

**Affiliations:** 1 Medicine, Rehman Medical Institute, Peshawar, PAK; 2 Accident and Emergency, District Headquarters Teaching Hospital, Dera Ismail Khan, PAK; 3 General Medicine, Rehman Medical Institute, Peshawar, PAK; 4 Cardiology, Northwest General Hospital and Research Center, Peshawar, PAK; 5 Cardiothoracic Surgery, Rehman Medical Institute, Peshawar, PAK

**Keywords:** post op vte, vte prophylaxis protocol, deep vein thrombosis (dvt), thromboembolism incidence, vte prophylaxis

## Abstract

Introduction: Venous thromboembolism (VTE) is a significant concern following orthopedic procedures, necessitating effective prophylactic measures. The rates of VTE prophylaxis, however, vary widely between institutions and nations, falling between 13% and 70% on average. In the absence of adequate thromboprophylaxis, pulmonary embolism, which accounts for 5% to 10% of mortality in hospitalized patients, is one of the leading causes of death. This study investigates the relationship between VTE prophylaxis and thromboembolism incidence, considering patient characteristics, risk assessment completion, anticoagulant prescription, and weight-bearing status.

Objectives: To evaluate the knowledge gap by examining the relationship between VTE prevention and the prevalence of thromboembolism also to examine the anticoagulant prescription trends for patients being discharged from orthopedic operations and to investigate the connection between post-procedure weight-bearing status and the development of VTE problems.

Methodology: A retrospective, cross-sectional design was employed, analyzing 200 orthopedic procedure patients from January to June of 2023 performed at Rehman Medical Institute. After getting ethical approval from the institutional ethical approval board, data were collected on the basis of variables encompassing patient attributes, surgery details, VTE risk factors, prophylaxis type, and VTE complications. Data was entered and analyzed via IBM SPSS Statistics for Windows, Version 22 (Released 2013; IBM Corp., Armonk, New York, United States), and the data were run through various tests including descriptive statistics, cross-tabulations, and chi-square. Results were then presented in the form of a table.

Results: Among 200 individuals 24 individuals experienced VTE, while 176 did not. Significant gender-based VTE differences were observed (p = 0.01). Hypertension (HTN) showed a significant association (p = 0.04) with VTE. Major surgeries were correlated with higher VTE incidence (p = 0.03). Pharmacological prophylaxis reduced the occurrence of VTE (p = 0.01). Early mobilization and Ted stockings were correlated with lower VTE incidence (p = 0.04, p = 0.12).

Conclusion: This study reveals gender-specific VTE differences, emphasizes the role of HTN, surgical nature, and prophylaxis in VTE incidence, and supports tailored prophylactic strategies. Our findings align with previous research and emphasize the significance of tailored prophylaxis strategies. By examining multiple factors, including gender, co-morbidities, surgical characteristics, and prophylactic methods, our study contributes to the evidence base that supports clinical decision-making and enhances patient safety in orthopedic surgery.

## Introduction

Patients undergoing orthopedic surgery are more likely to experience complications from VTE, and they represent a population where pharmacological thromboprophylaxis is necessary [1,2]. The rates of VTE prophylaxis, however, vary widely between institutions and nations, falling between 13% and 70% on average [3,4]. In the absence of adequate thromboprophylaxis, pulmonary embolism (PE), which accounts for 5% to 10% of mortality in hospitalized patients, is one of the leading causes of death [5]. Elective general surgery results in a 0.1% to 0.8% incidence of fatal PE, whereas elective hip replacement results in a 2% to 3% incidence, and hip fracture surgery results in a 4% to 7% incidence [6].

It is now well accepted that the pathophysiologic components of Virchow's triad from 1884 are connected to the onset of venous thromboembolism (VTE) [7]. Tourniquet use, immobility, surgical procedures harming blood vessels, trauma-induced release of clotting factors, and the use of certain substances like polymethylmethacrylate (PMMA) bone cement, which might exacerbate blood clotting tendencies, are some of these [8]. As a result, it is critical to place a high priority on VTE prevention and follow the suggested guidelines for these individuals.

Due to the treatable nature of the associated condition, pharmacological prophylaxis has become a standard procedure for patients undergoing major orthopedic surgery [9]. In orthopedic patients receiving routine VTE prophylaxis, fatal PE is rare, and rates of symptomatic VTE within three months vary from 1.3% to 10% [8]. To ensure the proper use of thromboprophylaxis in clinical practice, several organizations, including the American College of Chest Physicians, the American Academy of Orthopedic Surgeons, and the National Institute for Health and Care Excellence, have published evidence-based guidelines [10-13].

Although VTE prophylaxis is essential for minimizing thromboembolic events, research is still underway to determine the best strategy when it comes to orthopedic surgeries. The purpose of this study is to fill this knowledge gap by examining the relationship between VTE prevention and the prevalence of thromboembolism and also to investigate the connection between post-procedure weight-bearing status and the development of VTE problems. The findings will help to enhance patient safety, improve clinical procedures, and direct orthopedic surgical decision-making that is supported by the best available scientific evidence.

## Materials and methods

A retrospective, cross-sectional study design was employed to investigate the association between VTE prophylaxis and the incidence of thromboembolism following orthopedic procedures. The study population consisted of patients who had undergone orthopedic procedures from January to June of 2023. The study sample consisted of 200 patients. A systematic sampling method was used to ensure the sample size. The inclusion criteria included the patients who had completed and maintained medical records and who had consented to be part of the study. The exclusion criteria included patients who had incomplete medical records and who did not consent to be part of the study. After obtaining ethical approval from the institutional review board patient medical records from the orthopedic department were reviewed to identify eligible patients based on inclusion criteria. Data were collected from patient medical records to address the study objectives. The following variables were documented: patient characteristics: age, gender, admission and discharge dates, preoperative diagnosis, family history of VTE, previous history of VTE, operative procedures performed, completion of VTE risk assessment, VTE risk factors: obesity, smoking, previous history of thromboembolism, type of VTE prophylaxis: chemical or mechanical, anticoagulant prescribed on discharge, weight-bearing status after the procedure, details of VTE complications: location, severity, and management.

Data analysis (statistical methods)

Data were retrieved from the patient medical records of the orthopedics department at Rehman Medical Institute which were then entered in IBM SPSS Statistics for Windows, Version 22 (Released 2013; IBM Corp., Armonk, New York, United States). Descriptive statistics were used to summarize the collected data, including patient characteristics, VTE risk factors, and prophylaxis utilization. Statistical methods such as chi-square tests or logistic regression analysis were employed to assess the association between VTE prophylaxis and thromboembolism incidence. The significance level will be set at p < 0.05. Ethical approval was obtained from Rehman Medical Institute - Research Ethics Committee (RMI/RMI-REC/Article Approval/93 Nov 23,2023). Patient confidentiality and privacy were ensured by anonymizing the collected data. The study adhered to all applicable ethical guidelines and regulations.

## Results

The presented data outlines the characteristics and factors associated with the occurrence of VTE in a study cohort of 200 individuals. Among these participants, 24 individuals experienced VTE, while 176 did not. The analysis took into account variables such as gender, comorbidities, surgical nature, type of surgery, weight-bearing status, and pharmacological and mechanical VTE prophylaxis as seen in Table 1.

**Table 1:** Comparison of various factors between the two cohorts VTE: Venous thromboembolism; DM: Diabetes mellitus; HTN: Hypertension; CVA: Cerebrovascular accident; COPD: Chronic obstructive pulmonary disease; RA: Rheumatoid arthritis; Ca: Carcinoma *Major > 45 min, laparoscopic > 45 min, or arthroscopic **Minor < 45 min

Variable	Total (n = 200)	VTE (n = 24)	No VTE (n = 176)	p-value
Gender, n (%)				
Male	93 (46.5%)	14 (58.4%)	79 (44.9%)	0.01
Female	107 (53.5%)	10 (41.6%)	97 (55.1%)	
Comorbidities, n (%)				
DM	44 (22%)	3 (12.5%)	41 (23.3%)	0.23
HTN	61 (30.5%)	3 (12.5%)	58 (33%)	0.04
IHD	8 (4.0%)	2 (8.3%)	6 (34%)	0.24
CVA	2 (1.0%)	0 (0%)	2 (1.1%)	0.77
COPD	1 (0.5%)	0 (0%)	1 (0.5%)	0.88
RA	7 (3.5%)	0 (0%)	7 (3.9%)	0.40
Ca Breast	1 (0.5%)	1 (4.1%)	0 (0%)	0.71
Surgery Nature, n (%)				
Elective	129 (64.5%)	16 (66.7%)	113 (64.2%)	0.81
Emergency	71 (35.5%)	8 (33.3%)	63 (35.7%)	
Type of Surgery, n (%)				
Major*	155 (77.5%)	19 (79.1%)	136 (77.2%)	0.03
Minor**	45 (22.5%)	5 (20.9%)	40 (22.7%)	
Weight Bearing Status, n (%)				
Full Weight Bearing (FWB)	131 (65.5%)	13 (54.1%)	118 (67%)	0.21
Non-Full Weight Bearing (Non- FWB)	69 (34.5%)	11 (45.8%)	58 (32.9%)	
Pharmacological VTE Prophylaxis, n (%)	104 (52%)	7 (29.1%)	97 (55.1%)	0.01
Aspirin	74 (37%)	4 (16.6%)	70 (39.7%)	0.11
Apixaban	17 (8.5%)	2 (8.3%)	15 (8.5%)	0.43
Rivaroxaban	12 (6.0%)	1 (4.1%)	11 (6.2%)	0.95
Mechanical VTE Prophylaxis, n (%)				
Early Mobilization	198 (99%)	23 (94.8%)	175 (99.4%)	0.04
Ted Stockings	40 (20%)	2 (8.3%)	38 (21.5%)	0.12

Regarding gender, it was observed that 46.5% (n = 93) of male participants experienced VTE, compared to 53.5% (n = 107) of female participants. A statistically significant difference was found between the two genders in relation to VTE occurrence, with a p-value of 0.01. The influence of comorbidities on VTE was assessed, with specific conditions considered, including diabetes mellitus, hypertension (HTN), ischemic heart disease, cerebrovascular accident, chronic obstructive pulmonary disease (COPD), rheumatoid arthritis (RA), and carcinoma of the breast. While differences were observed in the incidence of VTE among individuals with various comorbidities, only HTN showed a statistically significant association (p = 0.04) with VTE occurrence. The study also examined surgical factors, including the nature of surgery (elective or emergency) and the type of surgery (major or minor). It was found that 66.7% of individuals who underwent elective surgery experienced VTE, compared to 33.3% in the emergency surgery group. Additionally, major surgeries were associated with a higher incidence of VTE (79.1%) compared to minor surgeries (20.9%). The p-value for the type of surgery was 0.03. The study also investigated the impact of pharmacological and mechanical VTE prophylaxis. Among the participants who received pharmacological prophylaxis, 29.1% experienced VTE, compared to 55.1% of those who did not (p = 0.01). Different agents were studied, including aspirin, apixaban, and rivaroxaban, with varying rates of VTE occurrence among users of these agents. Mechanical VTE prophylaxis methods were also assessed, with early mobilization and the use of Ted stockings examined. A lower incidence of VTE was observed among individuals who practiced early mobilization (94.8% vs. 99.4% for those who did not, p = 0.04). Similarly, individuals who used Ted stockings experienced a lower rate of VTE (5%) compared to those who did not (12%).

## Discussion

This study provides valuable insights into the factors influencing VTE incidence within a cohort of 200 individuals. By conducting a comprehensive analysis of various variables, including gender, comorbidities, surgical characteristics, weight-bearing status, and prophylactic measures, we aimed to uncover potential associations with VTE occurrence. Our findings reveal intriguing patterns and statistically significant relationships that warrant in-depth discussion and interpretation. In this section, we will delve into the implications of these observed trends, explore their alignment with existing literature, and examine their potential ramifications for clinical practice and future research endeavors.

Our study's outcomes both align with and extend the conclusions drawn from several prior investigations. For instance, the retrospective observational study by Nóbrega Catelas et al. examined VTE prophylaxis and incidence in patients undergoing total hip and knee replacements, highlighting a noteworthy reduction in symptomatic VTE rates post-implementation of prophylaxis measures, a trend consistent with our own observations [14]. Similarly, the emphasis placed by Majima et al. on the critical importance of preventing VTE after major orthopedic surgeries reinforces our study's focus on VTE prevention strategies [15].

In a broader context, the work of Wendelboe and Raskob underscores the global burden of thrombosis and its epidemiological aspects, providing a backdrop that accentuates the significance of our study's findings within the overall healthcare landscape [16]. Additionally, the exploration of VTE trends by Agarwal et al. following total shoulder arthroplasty sheds light on changes in VTE incidence and prophylaxis utilization, echoing our study's objectives [17]. Yoshida Rde et al.'s systematic review, which compares anticoagulants for VTE prophylaxis, contributes to the existing body of knowledge on prophylactic strategies, offering further context to our findings [18].

Further insights come from Coveney EI et al.'s evaluation of aspirin's role in VTE prophylaxis post-elective total hip arthroplasty, highlighting its effectiveness as a prophylactic agent [19]. Likewise, Shorr et al.'s discussion of antithrombotic agent selection for VTE prophylaxis in orthopedic surgery aligns with our examination of pharmacological prophylaxis options [20]. The comprehensive review by Flevas et al. provides an overview of thromboembolism prophylaxis methods, mirroring our study's aims and further strengthening the context for our findings [8].

Now turning to the interpretation of our own findings, we observe a statistically significant difference in VTE occurrence based on gender distribution, underscoring the importance of considering gender-related risk factors. The influence of comorbidities, particularly HTN, on VTE occurrence, echoes previous research that highlights the elevated risk associated with certain medical conditions. Notably, our analysis of surgical factors, including the type and nature of the surgery, reveals significant associations with VTE incidence, emphasizing the need for tailored prophylactic strategies based on surgical characteristics. Furthermore, the absence of a substantial difference in VTE occurrence based on weight-bearing status prompts a call for further exploration into this particular aspect.

When examining our evaluation of pharmacological VTE prophylaxis, we find alignment with previous research that underscores the efficacy of specific agents in reducing the risk of VTE. Additionally, our exploration of mechanical prophylaxis methods, such as early mobilization and the use of Ted stockings, reflects established strategies for preventing VTE. In a broader context, our study contributes to the growing body of evidence supporting the crucial role of comprehensive VTE prophylaxis strategies in orthopedic surgery, with potential implications for enhancing patient outcomes and informing future research directions.

While this study provides valuable insights into VTE prophylaxis and its outcomes, there are many limitations to consider. First, the study's retrospective design relies on data from patient records, which may contain incomplete or missing information, leading to potential bias. Second, the study was conducted at a single medical institute, which may limit the generalizability of the findings to a broader population. Additionally, the sample size was relatively small, and therefore a larger study is warranted. Another limitation was the study design which was a cross-sectional study.

Clinical Implications and future directions from the results of our study emphasize the importance of tailored VTE prophylaxis strategies based on patient and surgical characteristics, including gender, comorbidities, and surgical type. Furthermore, our findings highlight the potential benefits of both pharmacological and mechanical prophylaxis methods.

Moving forward, our study encourages further research into refining VTE risk assessment tools and exploring the role of extended prophylaxis. Comparative studies evaluating the effectiveness of various anticoagulants and prophylaxis regimens can contribute to evidence-based guidelines that enhance patient safety and improve clinical outcomes in orthopedic surgery.

## Conclusions

In conclusion, our retrospective, cross-sectional study provides valuable insights into the relationship between VTE prophylaxis and the incidence of thromboembolism following orthopedic procedures. Our findings align with previous research and emphasize the significance of tailored prophylaxis strategies. By examining multiple factors, including gender, co-morbidities, surgical characteristics, and prophylactic methods, our study contributes to the evidence base that supports clinical decision-making and enhances patient safety in orthopedic surgery.
